# Expression of sushi domain containing two reflects the malignant potential of gastric cancer

**DOI:** 10.1002/cam4.1793

**Published:** 2018-09-27

**Authors:** Shinichi Umeda, Mitsuro Kanda, Takashi Miwa, Haruyoshi Tanaka, Chie Tanaka, Daisuke Kobayashi, Masaya Suenaga, Norifumi Hattori, Masamichi Hayashi, Suguru Yamada, Goro Nakayama, Michitaka Fujiwara, Yasuhiro Kodera

**Affiliations:** ^1^ Department of Gastroenterological Surgery (Surgery II) Nagoya University Graduate School of Medicine Nagoya Japan

**Keywords:** expression, gastric cancer, hepatic recurrence, prognosis, *SUSD2*

## Abstract

Hepatic recurrence of gastric cancer (GC) is uncontrollable. Discovery of causative oncogenes and the development of sensitive biomarkers to predict hepatic recurrence are required to improve patients’ outcomes. In this study, recurrence pattern‐specific transcriptome analysis of 57 749 genes was conducted to identify mRNAs specifically associated with hepatic metastasis of patients with stage III GC who underwent curative resection. GC cell lines were subjected to mRNA expression analysis, PCR array analysis, and siRNA‐mediated knockdown. The expression levels of primary cancer tissues from 154 patients with resectable GC were determined and correlated with clinicopathological variables. Among 21 genes significantly overexpressed specifically in patients with hepatic recurrence, Sushi domain containing 2 (*SUSD2*) was selected as a promising target. PCR array analysis revealed that *SUSD2* mRNA levels positively correlated with those of *FZD7*,* CDH2*,* TGFB1*,* SPARC*,* ITGA5*, and *ZEB1*. Functional analysis revealed that knockdown of *SUSD2* significantly reduced the proliferation, migration, and invasiveness GC cell lines. Patients with high *SUSD2* expression were more likely to experience shorter disease‐free and overall survival. Analysis of the relation between disease recurrence pattern and *SUSD2* levels revealed that significantly more patients with hepatic metastases expressed higher levels of *SUSD2* mRNA. The cumulative incidence of hepatic recurrence was greater in patients with high *SUSD2* expression. In conclusion, *SUSD2* likely contributes to the malignant potential of GC and may serve as a novel biomarker that predicts hepatic recurrence after curative resection.

## 1 INTRODUCTION

Despite an overall decline in incidence over the last several decades,^1^ gastric cancer (GC) is the third and fifth leading cause of cancer‐related deaths in males and females, respectively, worldwide.^2^ Regardless of improvements in multimodal management strategies, approximately 40%‐80% of patients relapse,^3^ and despite the precision of curative resection, micrometastases remain outside the stomach and cause recurrence. Particularly, hepatic relapse contributes to the high incidence of GC‐related fatalities and represents a frequent and crucial problem for oncologists.^4^, ^5^ To address this serious problem, we require novel, sensitive biomarkers that predict hepatic recurrence and serve as targets to prevent or treat hepatic recurrence. A better understanding of the molecular mechanisms of GC progression is essential for developing such clinical tools. For example, advances in basic molecular oncological research led to the development of trastuzumab (anti‐HER‐2/neu antibody), which benefits many patients suffering from GC.^6^

To select candidate genes associated with the progression and metastasis of GC, we conducted a recurrence, pattern‐specific transcriptome analysis of 57 749 genes of patients with stage III GC who underwent curative resection. We identified sushi domain containing 2 (*SUSD2*) as a candidate oncogene that was associated with hepatic recurrence. To assess the significance of *SUSD2* in recurrent GC, we used GC cell lines and clinical samples to conduct detailed studies of *SUSD2* expression, function, as well as the clinical significance of *SUSD2* overexpression.

## 2 MATERIALS AND METHODS

### 2.1 Ethics

This study conformed to the ethical guidelines of the World Medical Association Declaration of Helsinki, Ethical Principles for Medical Research Involving Human Subjects and was approved by the Institutional Review Board of Nagoya University, Japan.

### 2.2 Transcriptome analysis

We conducted a recurrence, pattern‐specific transcriptome analysis of 57 749 genes to identify candidates specific to patients with stage III GC with hematogenous metastasis who underwent curative resection followed by S‐1 adjuvant therapy. For this purpose, we used the HiSeq platform (Illumina, San Diego, CA) to analyze primary GC tissues and their respective corresponding noncancerous adjacent gastric mucosa.^7^

### 2.3 Sample collection

The GC cell lines MKN1, MKN7, MKN45, MKN74, NUGC2, NUGC3, NUGC4, IM95, OCUM1, and SC‐6‐JCK cell lines were obtained from the Japanese Collection of Research Bio Resources Cell Bank (JCRB; Osaka, Japan). The AGS, KATOIII, and N87 cell lines were obtained from the American Type Culture Collection (Manassas, VA), and the GCIY cell line was obtained from Tohoku University (Miyagi, Japan). Cells were cultured at 37°C in Dulbecco's modified Eagle's medium (DMEM; Sigma‐Aldrich, St. Louis, MO, USA) supplemented with 10% fetal bovine serum in an atmosphere containing 5% CO_2_. Cell lines were analyzed using the short tandem repeat‐polymerase chain reaction (PCR) method and authenticated by the JCRB Cell Bank during June 2015.^8^

Primary GC tissues and the corresponding noncancerous adjacent tissues were collected from 154 patients who underwent gastric resection for GC at the Department of Gastroenterological Surgery, Nagoya University Hospital between 2001 and 2014. Written informed consent for the use of clinical samples and data, as required by the institutional review board, was obtained from all patients. The tissue samples were immediately frozen in liquid nitrogen and stored at −80°C. Since 2010, specimens have been histologically classified according to the 7th edition of the Union for International Cancer Control (UICC) classification system. Patients recruited before 2010 were reclassified accordingly. Since 2006, adjuvant chemotherapy using S‐1 (a fluorinated pyrimidine)^9^ has been orally administered to all patients with UICC stages II/III GC, unless contraindicated by a patient's condition.^10^

### 2.4 Analysis of *SUSD2* mRNA levels

*SUSD2* mRNA levels in cell lines and clinical samples were determined using a quantitative real‐time reverse‐transcription PCR (qRT‐PCR) assay. Total RNAs (10 µg per sample) were used to generate cDNAs that were amplified with primers specific for *SUSD2* (Table S1) as follows: initial denaturation at 95°C for 10 minutes, 40 cycles at 95°C for 10 seconds, and 60°C for 30 seconds. Samples were tested in triplicate, and samples without template were included in each PCR plate as negative controls. The ABI StepOnePlus Real‐Time PCR System (Applied Biosystems, Foster City, CA, USA) was used for real‐time detection of the emission intensity of SYBR‐Green fluorescence. Glyceraldehyde‐3‐phosphate dehydrogenase (*GAPDH*) mRNA served as an internal standard, and the expression level of each sample was calculated as the value of *SUSD2* mRNA divided by that of *GAPDH* mRNA.^11^

### 2.5 PCR array analysis

To identify genes coordinately expressed with *SUSD2* in GC cell lines, we used the Human Epithelial to Mesenchymal Transition (EMT) RT^2^ Profiler PCR Array (Qiagen, Hilden, Germany). This array includes 84 “key” genes that encode proteins with the functions as follows: transcription factor, extracellular matrix protein as well as proteins involved in the epithelial‐mesenchymal transition (EMT), cell differentiation, morphogenesis, growth, proliferation, migration, cytoskeleton, and signaling pathways.^7^

### 2.6 Small interfering RNA (siRNA)‐mediated knockdown of *SUSD2*

We designed four siRNAs specific for *SUSD2* (*SUSD2*‐siRNA). MKN1 and AGS cells were cultured in 24‐well plates (5 × 10^4^ cells/mL). Cells were transiently transfected the next day with 100 nmol/L siRNAs specific for *SUSD2* (*SUSD2*‐siRNA; Table S1) or a control siRNA (siControl). A NEON electroporation system (Invitrogen, Massachusetts, USA) was used to introduce the siRNAs into cells. MKN1 cells were subjected to a pulse voltage = 1400 V, pulse width 20 ms (two pulses), and AGS cells were subjected to a pulse voltage = 1500 V, pulse width 10 ms (three pulses). Knockdown efficiency was determined using qRT‐PCR 24 hours after transfection and Western blotting analysis 72 hours after transfection. Western blotting analysis using a mouse anti‐SUSD2 polyclonal antibody (ab168162; Abcam, Cambridge, UK) diluted 1:100 were performed as previously described.^12^ Cells were cultured in RPMI medium without antibody for 72 hours and then used for functional assays.

### 2.7 Assays of cell proliferation, migration, and invasion

Briefly, cell proliferation was evaluated using the Cell Counting Kit‐8 (Dojindo Molecular Technologies, Inc, Kumamoto, Japan). Cells (5 × 10^3^ cells per well) were incubated, and the optical density of the solution in each well was measured on days 1, 3, 5, 7 after the addition of 10 µL of Cell Counting Kit‐8 solution. The ability of GC cells to invade Matrigel was determined using BioCoat Matrigel invasion chambers (BD Biosciences, Bedford, MA) according to the manufacturer's protocol. Cells (2.5 × 10^4^) in serum‐free DMEM were added to each upper well of the chamber. After 44 hours, cells on the lower surface of the membrane were fixed, stained, and a microscope (200 × magnification) was used to count the cells in eight randomly selected fields. Cell migration was evaluated using wound‐healing assays as previously described. The width of the wound was measured at 100‐mm intervals measurements per well, 40 × magnification.

### 2.8 Evaluation of the clinical significance of *SUSD2* expression

*SUSD2* mRNA levels were determined in 154 matched pairs of resected gastric tissues from patients with stage I, II, or III GC to determine the risk of recurrence after curative resection. Patients were stratified into high or low expression groups (greater or lower than the median *SUSD2* value of GC tissues, respectively). For external validation of the survival data, we accessed a public‐domain integrated dataset comprising 1065 patients with GC from three major cancer research centers (Berlin, Bethesda, and Melbourne; https://kmplot.com/analysis/).

### 2.9 Statistical analysis

Differences in the values of qualitative variables were compared between groups using the chi‐square test, and quantitative variables were compared using the Mann‐Whitney test. The significance of the difference between two variables was assessed using Spearman's rank correlation coefficient. Overall and disease‐free survival rates were calculated using the Kaplan‐Meier method, and the difference between survival curves was analyzed using the log‐rank test. Risk factors for recurrence and survival were assessed using the Cox hazards ratio model. *P* < 0.05 was considered statistically significant. Statistical analyses were performed using JMP 13 software (SAS Institute Inc, Cary, NC, USA).

## 3 RESULTS

### 3.1 Identification of candidate markers

We identified 21 candidate markers that were specifically expressed at significantly higher levels in hepatic metastatic GC tissues (Table 1). We chose to pursue a study of *SUSD2* for the reasons as follows: (a) association with gastric cancer progression (*SUSD2* is a membrane protein that may mediate interactions between cells and between cells and cell‐matrix adhesion molecules.), (b) no report of an association of *SUSD2* expression with GC, (c) results of a pilot study of GC cell lines (Shinichi Umeda (US), Mitsuro Kanda (MK), Takashi Miwa (TM), Haruyoshi Tanaka (HT), Chie Tanaka (CT), Daisuke Kobayashi (DK), Masaya Suenaga (MS), Norifumi Hattori (NH), Masamichi Hayashi (MH), Suguru Yamada (SY), Goro Nakayama (GN), Michitaka Fujiwara (MF), Yasuhiro Kodera (YK)).

**Table 1:** List of candidate genes upregulated in gastric cancer tissues from patients with hepatic recurrence not with peritoneal and lymph node recurrence

Symbol	H‐rec/Non‐rec		Full name	Location	Function	P‐rec/Non‐rec		N‐rec/Non‐rec	
	Log_2_	*P* value				Log_2_	*P* value	Log_2_	*P* value
*SUSD2*	2.976	<0.001	Sushi domain containing 2	22q11.23	Cytokine receptor	0.464	0.4	0.302	0.583
*GAL*	4.278	<0.001	Galanin and GMAP prepropeptide	11q13.2	Endocrine hormone of nervous systems	2.076	0.11	1.34	0.192
*COMP*	4.187	<0.001	Cartilage oligomeric matrix protein	19p13.11	Extracellular matrix protein	0.76	0.452	1.173	0.075
*IGSF1*	3.546	<0.001	Immunoglobulin superfamily member 1	Xq26.2	Immunoglobulin	−0.899	1	0.601	1
*BCAM*	2.123	<0.001	Basal cell adhesion molecule	19q13.32	Laminin receptor	−0.554	0.269	0.042	0.933
*ASGR2*	3.56	<0.001	Asialoglycoprotein 2	17p13.1	Mediator of endocytosis of glycoproteins	−0.124	1	0.452	1
*RNF182*	5.362	<0.001	Ring finger protein 182	6p23	Mediator of MHC‐I antigen	−0.124	1	2.317	1
*CYP2W1*	6.809	<0.001	Cytochrome P450 family 2 subfamily W member 1	7p22.3	Metabolic enzyme	1.45	0.138	1.667	0.12
*FABP3*	3.774	<0.001	Fatty acid binding protein 3	1p35.2	Metabolic enzyme	0.051	0.953	0.992	0.229
*TKTL1*	6.109	<0.001	Transketolase like 1	Xq28	Metabolic enzyme	−2.08	1	−2.758	1
*GPC3*	2.99	<0.001	Glypican 3	Xq26.2	Multifunction membrane protein	−0.997	0.151	0.465	0.491
*TCF7L1*	2.288	<0.001	Transcription factor 7 like 1	2p11.2	Regulator of cell cycle	−0.26	0.651	−0.386	0.497
*HIF3A*	4.168	<0.001	Hypoxia‐inducible factor 3 alpha subunit	19q13.32	Regulator of hypoxia‐inducible genes	0.29	0.717	−0.018	0.979
*MYO18B*	4.731	<0.001	Myosin XVIIIB	22q12.1	Regulator of muscle structure	4.325	0.094	−0.66	1
*TNNT1*	3.316	<0.001	Troponin T1, slow skeletal type	19q13.42	Regulator of muscle structure	1.675	0.067	−0.637	0.355
*RBP4*	3.549	<0.001	Retinol binding protein 4	10q23.33	Retinol carrier	−1.186	0.196	0.834	0.253
*PRSS1*	4.203	<0.001	Protease, serine 1	7q34	Serine protease	0.122	0.906	0.952	0.361
*GATA5*	2.944	<0.001	GATA binding protein 5	20q13.33	Transcriptional factor	−1.401	0.096	−0.398	0.554
*HIC2*	3.434	<0.001	HIC ZBTB transcriptional repressor 2	22q11.21	Transcriptional factor	0.523	0.352	0.843	0.135
*HMGA2*	3.291	<0.001	High mobility group AT‐hook 2	12q14.3	Transcriptional factor	0.421	0.535	0.612	0.343
*SMTNL2*	4.739	<0.001	Smoothelin like 2	17p13.2	Unknown	−0.879	0.338	1.108	0.227

H‐rec, hepatic recurrence; Non‐rec, no recurrence; P‐rec. Peritoneal recurrence; L‐rec, lymph node recurrence.

### 3.2 Expression of *SUSD2* in GC cell lines

**Figure 1:**
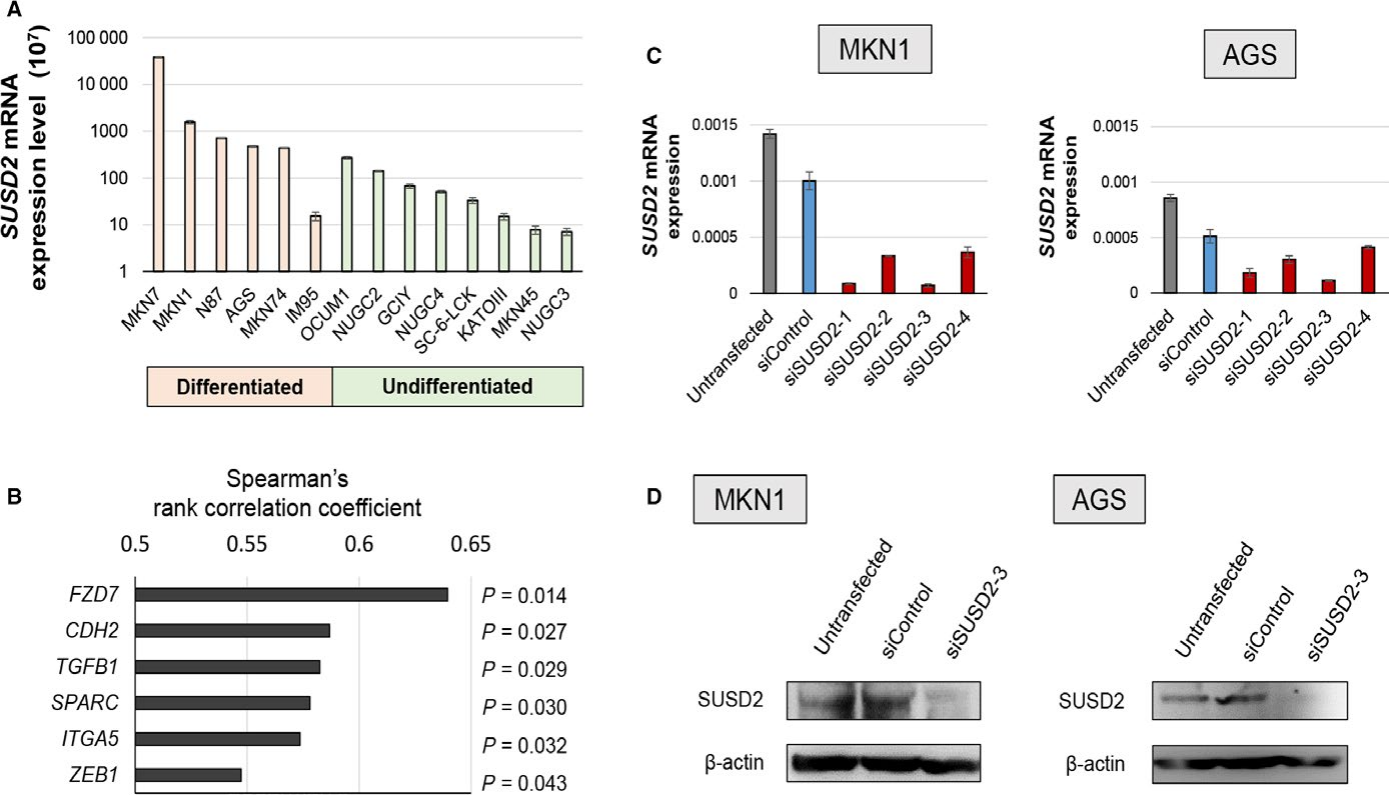
Analysis of *SUSD2* mRNA levels of gastric cancer (GC) cell lines, cancer‐related genes expressed cooperatively with SUSD2, and knockdown efficacy. A, *SUSD2* mRNA levels in GC cell lines. B, *SUSD2* mRNA and mRNAs of genes expressed at similar differential levels were identified using PCR array analysis. Spearman's rank correlation coefficient between the mRNA expression levels of *SUSD2* and those of *FZD7*,* CDH2*,* TGFB1*,* SPARC*,* ITGA5,* and *ZEB1* are shown. C, siRNA‐mediated *SUSD2*‐knockdown efficacy in MKN1 and AGS cell lines was determined using qRT‐PCR analysis. D, *SUSD2*‐knockdown efficacy was also determined using Western blotting analysis

*SUSD2* mRNA levels varied among GC cell lines. Differentiated GC cell lines expressed higher levels of *SUSD2* mRNA compared with those of undifferentiated GC cell lines. Cell lines established from metastatic sites in the liver, such as MKN1 and MKN45 cells, expressed the highest levels of *SUSD2* mRNA (Figure 1A).

### 3.3 PCR array analysis

There is little evidence that *SUSD2* contributes to the mechanism that regulates hematogenous metastasis associated with the epithelial‐mesenchymal transition (EMT). Therefore, we conducted a PCR array analysis to identify cancer‐related genes expressed coordinately with *SUSD2* with the aim of acquiring evidence to implicate *SUSD2* in cancer progression. We found that mRNAs encoding *FZD7*,* CDH2*,* TGFB1*,* SPARC*,* ITGA5*, and *ZEB1* were expressed at levels corresponding to those of *SUSD2* (Figure 1B).

### 3.4 Effect of *SUSD2* knockdown on the malignant phenotype of GC cells

We selected MKN1 and AGS cells for subsequent analyses, because they expressed the second and fourth highest levels of *SUSD2* mRNA *SUSD2* mRNA expression levels in MKN7 and N87 cells were high; however, these cells were not analyzed, because they were unsuitable for the invasion assay based on our pilot experiments (unpublished data). qRT‐PCR analysis revealed that si*SUSD2*‐3 yielded the highest level of inhibition (Figure 1C). The knockdown efficacy of si*SUSD2*‐3 was confirmed by Western blotting analysis (Figure 1D).

**Figure 2:**
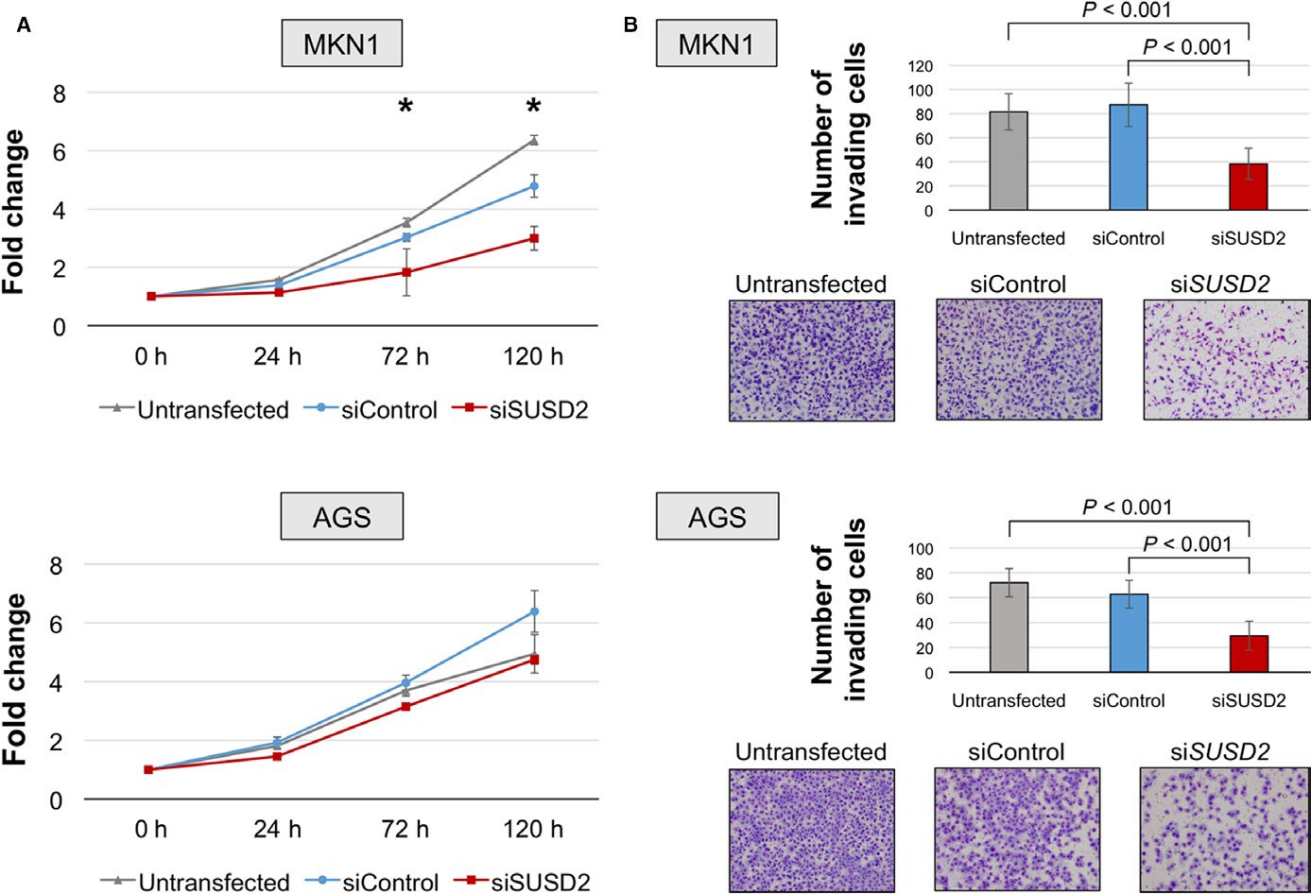
Cell proliferation and invasion assays. A, Cell proliferation assay. Inhibition of *SUSD2* expression significantly decreased the proliferation of MKN1and AGS cells. *P < 0.05. B, Cell invasion assays. The number of invading cells was significantly lower in cells transfected with the *SUSD2*‐siRNA

**Figure 3:**
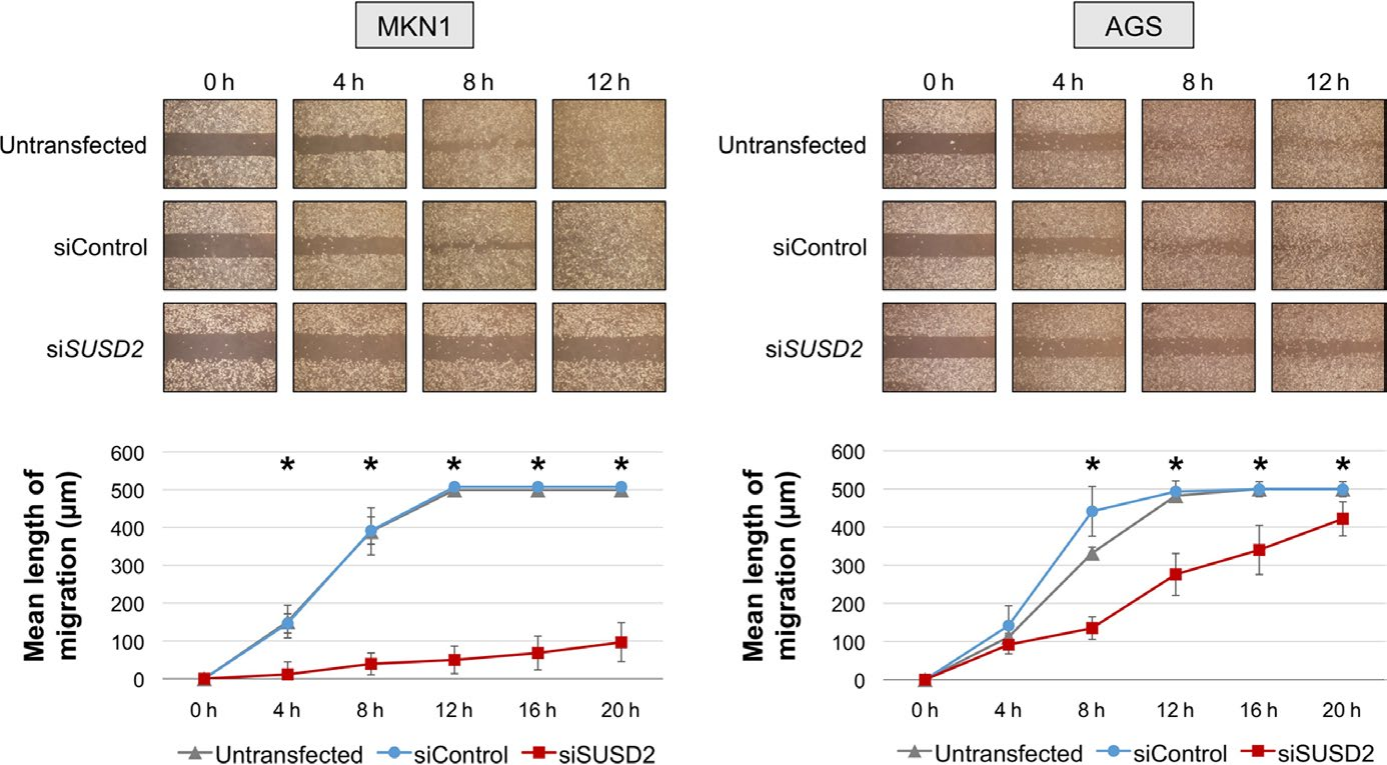
Cell migration assay. The migration of MKN1 and AGS cells transfected with the *SUSD2‐*sRNA was significantly decreased vs control cells. *P < 0.05

We next determined the effects of si*SUSD2*‐3 on cell proliferation, invasion, and migration. Inhibition of *SUSD2* expression significantly decreased the proliferation of MKN1 cells (40% and 37% decreases on days 3 and 5, respectively) and slightly decreased the proliferation of AGS cells (Figure 2A). Further, si*SUSD2* inhibited the invasion of Matrigel by MKN1 and AGS cells by 44% 46%, respectively, compared with untransfected cells (Figure 2B). si*SUSD2* inhibited the migration of MKN1 and AGS cell by 10% and 57%, respectively, compared with untransfected cells 12 hours after transfection (Figure 3).

### 3.5 Clinical significance of *SUSD2* expression

**Figure 4:**
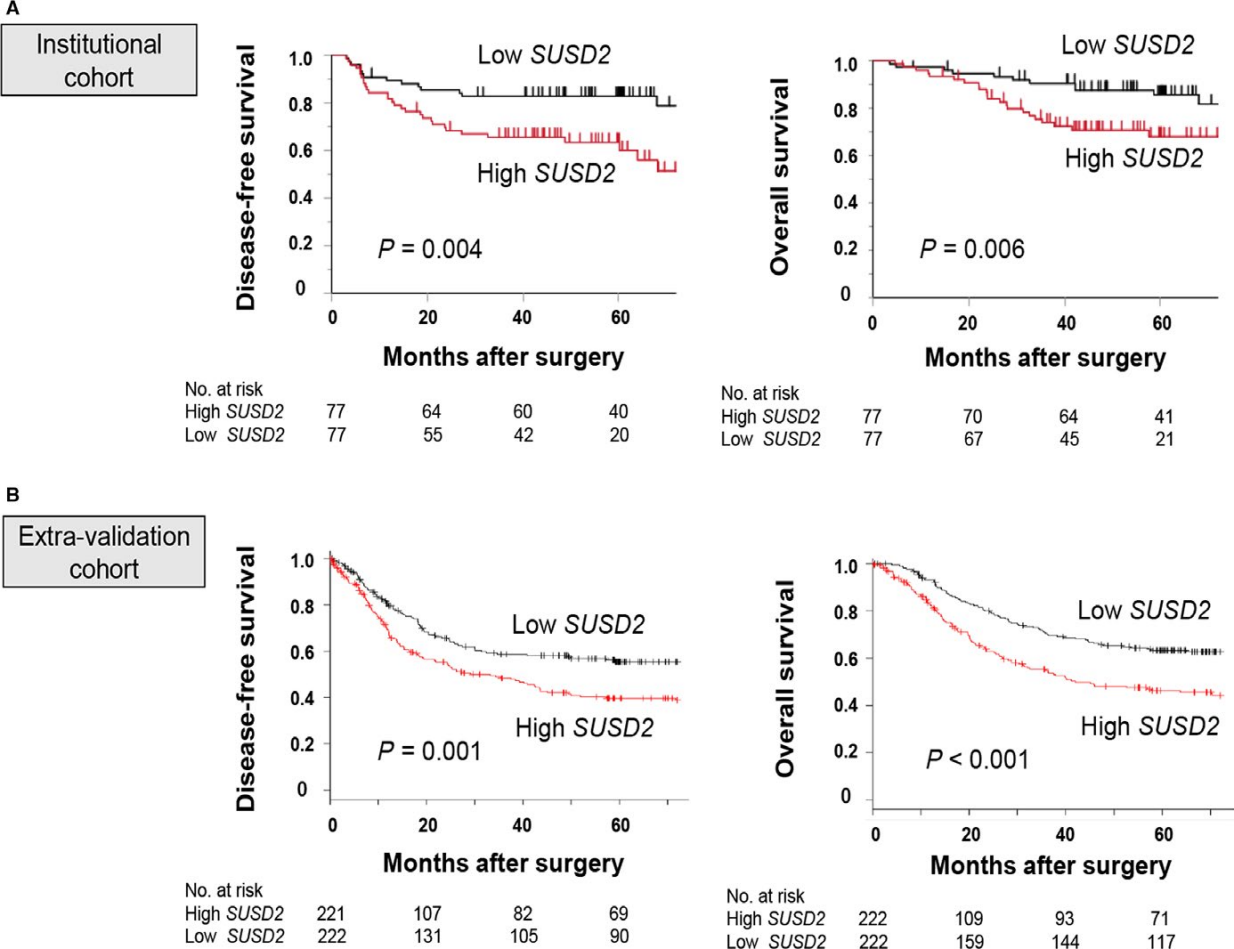
Prognostic implications of *SUSD2* mRNA expression in patients with GC after curative resection. A, Kaplan‐Meyer analysis of disease‐free survival and overall survival. B, Kaplan‐Meyer analysis of disease‐free survival and overall survival of patients in the external‐validation cohort

The patient population included 114 males and 40 females aged 26‐96 years (65.7 ± 10.6 years, mean ± SD), and 83 and 71 patients were diagnosed with undifferentiated or differentiated GC, respectively. According to the 7th edition of the UICC classification, 46, 40, and 68 patients were in stages I, II, and III, respectively. High *SUSD2* expression significantly associated with differentiation but not with tumor depth, invasive growth, lymph node metastasis, lymphatic involvement, vessel invasion, or tumor stage (Table S2). Patients in the high group were more likely to experience shorter disease‐free survival and overall survival (Figure 4A). Similar results were acquired using the external‐validation cohort (Figure 4B). Multivariable analysis identified high *SUSD2* mRNA levels as an independent prognostic factor for recurrence of patients with resected GC (hazard ratio, 2.89; 95% confidence interval, 1.52‐5.82; *P* = 0.001; Table 2). Further, *SUSD2* mRNA levels were an independent prognostic factor of overall survival (hazard ratio, 3.13; 95% confidence interval, 1.53‐6.82; *P* = 0.002; Table S3).

**Table 2:** Prognostic factors for disease‐free survival of 154 patients

	Univariate			Multivariable		
	Hazard ratio	95% CI	*P* value	Hazard ratio	95% CI	*P* value
Age (≥65)	1.13	0.62‐2.12	0.679			
Gender (male)	0.84	0.45‐1.66	0.606			
Tumor location (Lower)	0.84	0.44‐1.55	0.584			
Tumor multiplicity	0.47	0.08‐1.54	0.247			
Tumor size (≥60 mm)	2.45	1.35‐4.44	0.004	1.87	1.02‐3.44	0.044*
Carcinoembryonic antigen (>5 ng/mL)	1.67	0.75‐3.33	0.194			
Carbohydrate antigen 19‐9 (>37 IU/mL)	2.73	1.34‐5.17	0.007	1.75	0.84‐3.46	0.132
Tumor depth (pT4)	2.98	1.64‐5.45	<0.001	1.92	1.03‐3.64	0.041*
Lymph node metastasis	14.6	5.28‐60.3	<0.001	7.26	2.42‐32.3	<0.001*
Tumor differentiation (undifferentiated)	1.51	0.83‐2.86	0.179			
Lymphatic involvement	2.73	1.35‐5.17	0.007	1.72	0.27‐33.6	0.607
Vascular invasion	4.32	2.17‐9.58	<0.001	2.27	1.08‐5.45	0.029*
Postoperative adjuvant chemotherapy	1.65	0.91‐2.99	0.10			
High *SUSD2* expression	2.47	1.33‐4.81	0.004	2.89	1.52‐5.82	0.001*

a Statistically significant in multivariable analysis. CI, confidence interval; UICC, Union for International Cancer Control.

**Figure 5:**
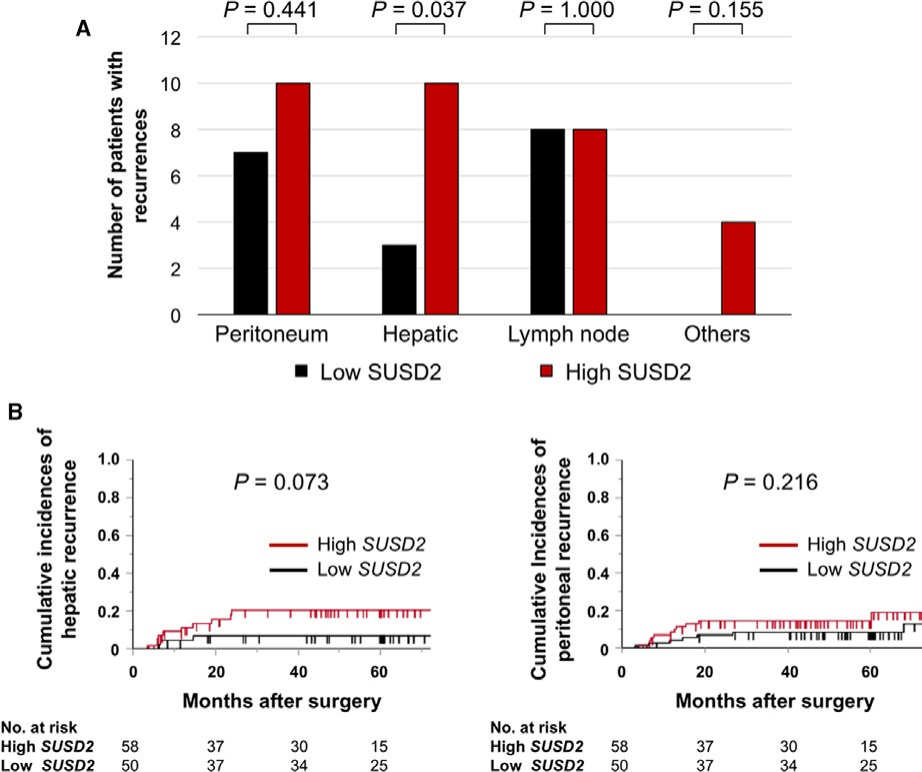
Analysis of recurrence patterns. A, Numbers of sites of initial recurrence in the high and low expression groups. B, Cumulative incidence of hepatic and peritoneal recurrence

Of all 154 patients, 44 patients (28.6%) experienced recurrence and there were a total of 50 initial relapse sites. Analysis of the association of initial recurrence patterns and *SUSD2* mRNA levels revealed that significantly more patients were included in the high group (*P* = 0.037; Figure 5A). The cumulative occurrence of hepatic recurrence was greater in the high group, in contrast to patients with peritoneal recurrence (Figure 5B).

## 4 DISCUSSION

Here we used transcriptome analysis to identify *SUSD2* mRNA levels as a candidate marker of hepatic recurrence and survival of patients with GC who underwent curative resection. Human *SUSD2* was identified in a cDNA library enriched for genes that encode membrane and secreted proteins that are highly expressed in cancer cells, but at low levels in normal tissues.^13^ *SUSD2* resides on chromosome 22q11.23, comprises fifteen exons, and encodes a type I membrane protein (822 amino acid residues). The predicted SUSD2 amino acid sequence comprises a somatomedin B and adhesion‐associated domains present in MUC4 and other proteins (AMOP) as well as von Willebrand factor type D and sushi domains. The latter play significant roles in mediating intercellular and cell‐to‐matrix adhesion.^14^ As predicted by its transmembrane domain, SUSD2 localizes to the plasma membrane in vivo.^15^

Evidence indicates that *SUSD2* contributes to oncogenesis. For example, *SUSD2* increases the invasiveness of breast cancer cells and may act as a component of the mechanism of immune evasion through induction of apoptosis of the Jurkat T cell line.^14^ *SUSD2* recruits macrophages into the tumor microenvironment, and promotes M2 polarization, indicating that inhibiting the function of *SUSD2* may serve as an effective therapy for patients with breast cancer.^16^

In contrast, *SUSD2* may act as a tumor suppressor. For example, *SUSD2* exhibits tumor suppressor activity in high‐grade serous ovarian carcinomas^17^ and may function as a tumor suppressor in renal cell carcinoma and lung cancer.^18^ Low levels of *SUSD2* expression correlate with the aggressive behavior of non‐small cell lung cancer cells.^19^ *SUSD2* is expressed in endometrial carcinoma cells, and suppressing its expression following treatment with TGFβ or a specific siRNA increases apoptosis and senescence. The present study presents the first evidence, to our knowledge, that *SUSD2* acts as an oncogene in GC.^20^

We show here that inhibiting *SUSD2* mRNA expression using a specific siRNA inhibits cell proliferation, invasion, and migration, indicating that *SUSD2* is required to establish the malignant phenotype of GC cells. To address this issue in more detail, we performed PCR array analysis and found that *FZD7*,* CDH2*,* TGFB1*,* SPARC*,* ITGA5,* and *ZWB1* mRNAs were overexpressed in concert with *SUSD2*. These findings implicate the contribution of the WNT signaling pathway to the malignant phenotype of GC. Specifically, the evolutionarily conserved WNT signaling pathway controls intercellular interactions during embryogenesis, and dysfunction of this pathway is implicated in a spectrum of human diseases, particularly solid and hematologic malignancies.^21^ Thus, WNT signaling may serve as a target of cancer therapy.^22^ Fzd7 is the WNT receptor most commonly upregulated in diverse cancers and plays a significant role in stem cell biology and cancer development and progression. Small molecules that act as classic GPCR modulators targeting Fzd7 to regulate WNT/β‐catenin signaling may therefore represent potential cancer therapeutics.^23^

The EMT involves the conversion of epithelial cells to migratory and invasive cells, and the activation of the EMT is closely associated with the motility and invasiveness of GC cells.^24^, ^25^ *FZD7*,* CDH2*,* TGFB1*,* SPARC*,* ITGA5,* and *ZWB1* are associated with the EMT and malignant cell function, indicating that *SUSD2* may play a role in the WNT pathway and the EMT.^23^, ^26^, ^27^, ^28^, ^29^, ^30^ Therefore, *SUSD2* may serve as a therapeutic target for inhibiting inappropriate WNT signaling or induction of the EMT.

Although the levels of *SUSD2* mRNA were not significantly associated with clinicopathological variables that influence the malignant phenotype of GC (tumor size, lymph nodes metastasis, and UICC stage), *SUSD2* mRNA levels were closely associated with recurrence and survival after curative surgery. These results are consistent with those of our analysis of a validation cohort. Therefore, *SUSD2* mRNA level may reflect the malignant potential of GC independent of clinicopathological markers. Moreover, multivariable analysis revealed that *SUSD2* mRNA levels served as an independent risk factor of recurrence and death after complete resection of GC, indicating the utility of *SUSD2* expression as a novel predictor of prognosis.

To translate our findings into clinical practice, *SUSD2* mRNA levels, determined using biopsy or surgical specimens, might be useful to predict patients who are at high risk of relapse. Stronger adjuvant chemotherapy such as cisplatin or taxane, or neoadjuvant chemotherapy, may prevent recurrence.^31^, ^32^ Frequent follow‐up may facilitate an earlier diagnosis of recurrence, allowing immediate administration of chemotherapy and curative resection of the site of recurrence, which will likely improve prognosis.

The present study shows that *SUSD2* expression predicted recurrence and survival, as well as the recurrence pattern after curative resection of GC. It is important to note that high levels of *SUSD2* expression are closely associated with hematogenous metastasis vs lymphatic and peritoneal recurrence. Therefore, we recommend that patients with high *SUSD2* mRNA levels, which indicate hematogenous recurrence, undergo intensive preoperative and postoperative surveillance, such as with Gd‐EOB‐DTPA, enhanced magnetic resonance imaging of the liver,^33^ bone scintigraphy, and positron emission tomography for early detection.^34^ It is interesting to note an increase in the incidence of western‐type GC, which develops on the esophagogastric junction or upper stomach in the absence of *Helicobacter pylori* infection, and tends to recur via the hematogenous route.^31^, ^35^, ^36^ The prediction and control of hepatic recurrence are more important for these patients, lending weight to the potentially important clinical implications of our findings.

There are some limitations to this study. First, PCR array analyses identified mRNAs encoding proteins associated with the EMT or the WNT signaling pathway, although there is no evidence that *SUSD2* contributes to the activities of these pathways. Therefore, pathway analyses should be conducted to further understand the biological functions of *SUSD2* in GC. Second, this was a retrospective study of a small number patients treated at a single center. External validation using large cohorts from multiple institutions as well as bioinformatics analysis of large datasets are required to validate our present findings. In summary, our results indicate that *SUSD2* expression reflects the malignant potential of GC and will serve as novel biomarker that predicts recurrence and prognosis after curative resection of GC *SUSD2* may serve as a target of therapy and therefore will facilitate the development of effective therapeutic strategies.

## CONFLICTS OF INTEREST

None declared.
