# Supplementary material for: Expression of sushi domain containing two reflects the malignant potential of gastric cancer
Source: Cancer Med. 2018 Sep 27;7(10):5194–204. doi: 10.1002/cam4.1793 (PMC6198216; doi:10.1002/cam4.1793)
Supplement: Supplementary file 1 [file CAM4-7-5194-s001.doc]

|  | **Experiment** | **Type** | **Sequence (5´ - 3´)** | **Product size** | **Annealing temperature** |
| --- | --- | --- | --- | --- | --- |
| ***SUSD2*** | qRT-PCR | forward | GATGGAGAAGAGCGAGTTGG | 176bp | 60°C |
|  |  | reverse | TTGCAGTCCACTCCTGTGAG |  |  |
|  | siRNA | si*SUSD2*-1 | AGGUUUUGGGGUGAAAGUGAA |  | |
|  |  | si*SUSD2*-2 | UUUUUUGAAGGAAACAAGGCG |  |  |
|  |  | si*SUSD2*-3 | GCACCAACUUCACAUUCAA+dTdT |  |  |
|  |  | si*SUSD2*-4 | GGCUGUGCACCCCAACAAA+dTdT |  |  |
| ***GAPDH*** | qRT-PCR | forward | GAAGGTGAAGGTCGGAGTC | 226 bp | 60 °C |
|  |  | probe | CAAGCTTCCCGTTCTCAGCC |  |  |
|  |  | reverse | GAAGATGGTGATGGGATTTC |  |  |

**Table S1.　Sequences of primers and siRNAs**

*SUSD2, Sushi domain connecting 2*; *GAPDH, glyceraldehyde-3-phosphate dehydrogenase*;

qRT-PCR, quantitative real-time reverse-transcription polymerase chain reaction; bp, base pair.
