# Supplementary material for: Expression of sushi domain containing two reflects the malignant potential of gastric cancer
Source: Cancer Med. 2018 Sep 27;7(10):5194–204. doi: 10.1002/cam4.1793 (PMC6198216; doi:10.1002/cam4.1793)
Supplement: Supplementary file 2 [file CAM4-7-5194-s002.docx]

**Table S2. Association between expression level of *SUSD2* mRNA and clinicopathological parameters of 154 resected gastric cancer patients.**

| **Clinicopathological parameters** | **Low *SUSD2***  **in GC tissue (n)** | **High *SUSD2***  **in GC tissue (n)** | **P value** |
| --- | --- | --- | --- |
| Age  <65 year  ≥65 year | 26  51 | 37  40 | 0.100 |
| Gender  Male  Female | 55  22 | 59  18 | 0.500 |
| Tumor location  Lower  Other | 35  42 | 24  53 | 0.097 |
| Tumor multiplicity  Present  Absent | 9  68 | 3  74 | 0.130 |
| Tumor size (mm)  <60  ≥60 | 53  24 | 51  26 | 0.864 |
| Carcinoembryonic antigen (ng/ml)  ≤5  >5 | 63  14 | 67  10 | 0.505 |
| Carbohydrate antigen 19-9 (IU/ml)  ≤37  >37 | 65  12 | 64  13 | 1.000 |
| pT  T1, 2, 3  T4 | 58  19 | 47  30 | 0.083 |
| Lymph node metastasis  Absent  Present | 34  43 | 33  44 | 1.000 |
| Tumor differentiation  Differentiated  Undifferentiated | 43  34 | 28  49 | 0.023* |
| Lymphatic involvement  Absent  Present | 13  64 | 16  61 | 0.680 |
| Vascular invasion  Absent  Present | 38  39 | 34  43 | 0.628 |
| Pathological UICC stage  I  II  III | 27  17  33 | 19  23  35 | 0.307 |
| Postoperative adjuvant chemotherapy  Present  Absent | 31  46 | 28  49 | 0.740 |

*Statistically significant (P < 0.05). GC, gastric cancer; UICC, Union for International Cancer Control.
