# Supplementary material for: Expression of sushi domain containing two reflects the malignant potential of gastric cancer
Source: Cancer Med. 2018 Sep 27;7(10):5194–204. doi: 10.1002/cam4.1793 (PMC6198216; doi:10.1002/cam4.1793)
Supplement: Supplementary file 3 [file CAM4-7-5194-s003.docx]

**Table S3. 　Prognostic factors for overall survival of 154 patients.**

|  | **Univariate** | | | **Multivariable** | | |
| --- | --- | --- | --- | --- | --- | --- |
|  | **Hazard　ratio** | **95% CI** | **P value** | **Hazard ratio** | **95% CI** | **P value** |
| Age (≥ 65) | 0.86 | 0.44-1.71 | 0.680 |  |  |  |
| Gender (male) | 0.73 | 0.37-1.57 | 0.420 |  |  |  |
| Tumor location (Lower) | 0.63 | 0.29-1.28 | 0.207 |  |  |  |
| Tumor multiplicity | 0.60 | 0.10-1.96 | 0.443 |  |  |  |
| Tumor size (≥60 mm) | 3.44 | 1.76-6.92 | <0.001 | 3.62 | 1.79-7.60 | <0.001* |
| Carcinoembryonic antigen (>5 ng/ml) | 1.59 | 0.64-3.44 | 0.290 |  |  |  |
| Carbohydrate antigen 19-9 (>37 IU/ml) | 2.90 | 1.31-5.94 | 0.001 | 1.93 | 0.83-4.24 | 0.118 |
| Tumor depth (pT4) | 3.61 | 1.85-7.29 | <0.001 | 2.68 | 1.30-5.76 | 0.008* |
| Lymph node metastasis | 11.58 | 4.09-48.5 | <0.001 | 5.44 | 1.77-25.0 | 0.002* |
| Tumor differentiation (undifferentiated) | 3.44 | 1.76-6.92 | <0.001 |  |  |  |
| Lymphatic involvement | 9.88 | 2.12-175 | <0.001 | 0.93 | 0.12-19.6 | 0.953 |
| Vascular invasion | 5.18 | 2.31-13.8 | <0.001 | 3.51 | 1.41-10.6 | 0.005* |
| Postoperative adjuvant chemotherapy | 1.53 | 0.78-2.99 | 0.211 |  |  |  |
| High *SUSD2* expression | 2.64 | 1.32-5.63 | 0.006 | 3.13 | 1.53-6.82 | 0.002* |

*Statistically significant in multivariable analysis. CI, confidence interval; UICC, Union for International Cancer Control.
